# Circ_0015756 promotes the progression of ovarian cancer by regulating miR-942-5p/CUL4B pathway

**DOI:** 10.1186/s12935-020-01666-1

**Published:** 2020-11-27

**Authors:** Zhenhua Du, Lei Wang, Yu Xia

**Affiliations:** Department of Obstetrics and Gynecology, Shengjing Hospital of China Medical University, NO. 36 Sanhao Street, Heping District, Shenyang City, 110021 Liaoning Province China

**Keywords:** Ovarian cancer, Circ_0015756, miR-942-5p, CUL4B

## Abstract

**Background:**

Ovarian cancer (OC) is the gynecologic cancer with the highest mortality. Circular RNAs (circRNAs) play a vital role in the development and progression of cancer. This study aimed to explore the potential role of circ_0015756 in OC and its molecular mechanism.

**Methods:**

The levels of circ_0015756, microRNA-942-5p (miR-942-5p) and Cullin 4B (CUL4B) were determined by quantitative real-time PCR (qRT-PCR) or Western blot assay. Cell proliferation, apoptosis, migration and invasion were assessed by Cell Counting Kit-8 (CCK-8), colony formation assay, flow cytometry and transwell assay. The levels of proliferation-related and metastasis-related proteins were measured by Western blot assay. The relationship between miR-942-5p and circ_0015756 or CUL4B was verified by dual-luciferase reporter assay, RNA immunoprecipitation assay and RNA pull-down assay. Xenograft assay was used to analyze tumor growth in vivo.

**Results:**

Circ_0015756 and CUL4B levels were increased, while miR-942-5p level was decreased in OC tissues and cells. Depletion of circ_0015756 suppressed proliferation, migration and invasion and promoted apoptosis in OC cells. Down-regulation of circ_0015756 hindered OC cell progression via modulating miR-942-5p. Also, up-regulation of miR-942-5p impeded OC cell development by targeting CUL4B. Mechanistically, circ_0015756 up-regulated CUL4B via sponging miR-942-5p. Moreover, circ_0015756 silencing inhibited tumor growth in vivo.

**Conclusion:**

Knockdown of circ_0015756 suppressed OC progression via regulating miR-942-5p/CUL4B axis, suggesting that circ_0015756 might be a potential therapeutic target for ovarian cancer.

## Highlights

1. Circ_0015756 was up-regulated in OC tissues and cells.
2. Knockdown of circ_0015756 inhibited OC progression.
3. Circ_0015756 promoted OC progression via miR-942-5p/CUL4B axis.
4. Circ_0015756 depletion inhibited OC growth in vivo.

## Introduction

Ovarian cancer (OC) is a malignant tumor of the female reproductive system, and the mortality of epithelial OC ranks first among gynecological cancers [1]. The treatment of OC mainly includes surgery and platinum-based chemotherapy [2]. With the gradual aging of the population, the incidence of OC is increasing year by year [3]. Due to the lack of specific early clinical symptoms, OC is mostly diagnosed at advanced stage [4]. Therefore, exploring the molecular mechanism of OC progression to discover effective tumor markers is crucial for diagnosis and treatment of OC.

Circular RNAs (circRNAs) are a new type of endogenous RNAs with a covalent closed-loop structure without 5′-3′ polarity [5]. Mounting evidence has corroborated that circRNAs with complex tissue specificity participate in tumorigenesis and development through multiple pathways [6]. Furthermore, circRNAs can serve as promising therapeutic targets for gynecological cancers [7]. For example, circRHOBTB3 impeded OC development by inhibiting PI3K/AKT signaling pathway [8]. Additionally, hsa_circ_0009910 facilitated the malignant behaviors of OC via down-regulating microRNA-145 [9]. Also, hsa_circRNA_102958 aggravated the progression of OC by regulating microRNA-1205/SH2D3A pathway [10]. A previous research revealed that hsa_circ_0015756 derived from complement factor H (CFH) was significantly up-regulated in OC tissues [11]. Nonetheless, the biological function and potential basis of circ_0015756 in OC have not been investigated.

Compelling evidence has suggested that microRNAs (miRNAs) suppress gene expression by base-pairing with the 3′UTR of target mRNA [12]. Substantial studies have confirmed that miRNAs participate in many cellular processes, including cell differentiation, proliferation and metastasis [13]. For instance, miR-362-3p suppressed the metastasis of cervical cancer by inhibiting BCAP31 [14]. Moreover, miR-486-5p accelerated cell growth and mobility in endometrial carcinoma via repressing MARK1 [15]. Besides, Xie et al. [16] suggested that miR-942 level was conspicuously reduced in OC tissues. Nevertheless, the association between circ_0015756 and miR-942-5p remains unclear.

In this research, we verified that circ_0015756 was remarkably increased in OC. Furthermore, we clarified the function and potential mechanism of circ_0015756 in OC. These findings unveiled that circ_0015756 played a pro-oncogenic role in OC by regulating miR-942-5p/Cullin 4B (CUL4B) axis, which might provide a new biomarker for OC treatment.

## Materials and methods

### Specimen collection

OC tissues (n = 55) and adjacent normal tissues (n = 55) were obtained from 55 OC patients who underwent oophorectomy at Shengjing Hospital of China Medical University. Two histopathologists independently confirmed the results of all tissue samples. Inclusion criteria were: complete medical records; no treatment started before admission. Exclusion criteria were: recurrent OC; history of other malignancies; other clinical diseases. None of OC patients received any treatment before surgery. All participants signed written informed consent. The research was ratified by the Ethics Committee of Shengjing Hospital of China Medical University.

### Cell culture

Human normal ovarian epithelial cell line (IOSE80) was purchased from Shanghai and Shanghai Zhen Biotechnology Co., Ltd. (Shanghai, China). Two OC cell lines (OV90 and SKOV3) were commercially acquired from American Type Culture Collection (ATCC, Manassas, VA, USA). All cells were cultured in RPMI-1640 medium (cat no. SH30809.01; Hyclone, Logan, UT, USA) containing 10% fetal bovine serum (FBS, cat no. SH30084.03; Hyclone) with 5% CO_2_ at 37 °C.

### Cell transfection

Circ_0015756 small interfering RNA (si-circ_0015756) and negative control (si-NC), miR-942-5p mimics (miR-942-5p) and the control (miR-NC), miR-942-5p inhibitor (anti-miR-942-5p) and the control (anti-miR-NC), CUL4B overexpression vector (pcDNA-CUL4B) and negative control (pcDNA) were synthesized from GenePharma (Shanghai, China). Cell transfection was carried out using Lipofectamine 3000 (cat no. L3000-015; Invitrogen, Carlsbad, CA, USA) when cell confluence reached ~ 80%.

### Quantitative real-time PCR (qRT-PCR)

RNA was extracted from tissues and cells using Trizol (cat no. R0016; Beyotime, Shanghai, China). Next, the complementary DNA (cDNA) was synthesized using the specific reverse transcription kit (Takara, Dalian, China). Subsequently, the expression level was monitored using SYBR Premix Ex Taq (cat no. RR420A; Takara) and calculated using the 2^−ΔΔCt^ method. Glyceraldehyde 3-phosphate dehydrogenase (GAPDH) or U6 were identified as a loading control. The PCR amplification procedure included 95 °C for 10 min, followed by 40 cycles of 95 °C for 5 s, 60 °C for 10 s, and 72 °C for 10 s. The primers included: circ_0015756-F: 5′-TGGACGGAACCACCTCAATG-3′, circ_0015756-R: 5′-CCTGAAACCACCCTCACAAGT-3′; miR-942-5p-F: 5′-AGGGTCTTCTCTGTTTTGGC-3′, miR-942-5p-R: 5′-GTTGTGGTTGGTTGGTTTGT-3′; CUL4B-F: 5′-ACTCCTCCTTTACAACCCAGG-3′, CUL4B-R: 5′-TCTTCGCATCAAACCCTACAAAC-3′; GAPDH-F: 5′-GGGAAACTGTGGCGTGAT-3′, GAPDH-R: 5′-GAGTGGGTGTCGCTGTTGA-3′; U6-F: 5′-CTCGCTTCGGCAGCACA-3′, U6-R: 5′-AACGCTTCACGAATTTGCGT-3′.

### Cell viability assay

OV90 and SKOV3 cells were plated into 96-well plates at a density of 2 × 10^3^ cells/well after transfection with different combinations. After incubating at 37 °C for 0 h, 24 h, 48 h or 72 h, 10 μL of Cell Counting Kit-8 (CCK-8, cat no. C0038; Beyotime) solution was added to each well for 2 h. Then, the absorbance was measured at 450 nm using a Microplate Reader (cat no. CN-13055-50; BioTek, Burlington, VT, USA).

### Colony formation assay

The transfected OV90 and SKOV3 cells were injected into six-well plates and incubated for 2 weeks. After washing with phosphate-buffered saline (PBS, cat no. P1020; Solarbio, Beijing, China), the cells were fixed with 4% paraformaldehyde and stained with 0.5% crystal violet (cat no. C8470-25; Solarbio). Finally, the colonies were photographed and counted under a microscope.

### Western blot assay

Total protein was extracted with RIPA buffer (cat no. R0020; Solarbio). Subsequently, the protein samples were quantified using BCA Protein Assay Kit (cat no. ab102536; Abcam, Cambridge, UK) and then separated by 10% polyacrylamide gel electrophoresis (PAGE) and transferred onto PVDF membranes (cat no. P2938; Sigma, St Louis, MO, USA). After blocking with 5% non-fat milk for 2 h at room temperature, the membranes were incubated with primary antibodies against Ki67 (1:5000, cat no. ab92742; Abcam), CDK2 (1:2000, cat no. ab32147; Abcam), Snail (1:500, cat no. ab82846; Abcam), MMP9 (1:1000, cat no. ab38898; Abcam), CUL4B (1:2000, cat no. ab227724; Abcam) or β-actin (1:2000, cat no. ab8227; Abcam) overnight at 4 °C. After washing three times with TBST for 10 min each time, the membranes were probed with the corresponding HRP-labeled secondary antibody (1:20,000, cat no. ab205718; Abcam) for 2 h at room temperature. After the secondary antibody incubation, the membranes were rinsed three times with TBST for 10 min each time. Afterwards, the protein bands were examined using the ECL system (cat no. P0018AM; Beyotime).

### Flow cytometry

Cell apoptosis was evaluated using Annexin V-FITC Apoptosis Detection Kit (cat no. C1062M; Beyotime). The transfected OV90 and SKOV3 cells were washed with PBS (cat no. P1020; Solarbio) and then resuspended in binding buffer. Subsequently, the cells were stained with Annexin V-FITC and Propidium Iodide (PI) for 15 min. Finally, the apoptosis rate was assessed by FACScan Flow Cytometry (cat no. BD FACSCalibur 342975; BD Biosciences, San Diego, CA, USA).

### Transwell assay

The transfected OV90 and SKOV3 cells were injected into the upper chamber (cat no. 3422; Corning, Corning, NY, USA). At the same time, 10% FBS (cat no. SH30084.03; Hyclone) was injected into the lower chamber as attractant. After culturing for 24 h, the cells were fixed with methanol and stained with 0.1% crystal violet (cat no. C8470-25; Solarbio). Then, the migrated cells were counted under a microscope at 100× magnification. The difference in cell invasion test was that transwell was pre-coated with Matrigel (cat no. 354234; Corning).

### Dual-luciferase reporter assay

The wild-type luciferase reporter (circ_0015756-WT or CUL4B 3′UTR-WT) was formed via cloning circ_0015756 or CUL4B 3′UTR containing miR-942-5p binding site into pmirGLO vector (cat no. LM-1439; LMAI Bio, Shanghai, China). The mutant luciferase reporter (circ_0015756-MUT or CUL4B 3′UTR-MUT) was formed via mutating the binding site. Afterwards, the corresponding reporter and miR-NC or miR-942-5p were co-transfected into OV90 and SKOV3 cells. The Firefly and Renilla luciferase activities were measured via Dual-Lucy Assay Kit (cat no. D0010; Solarbio). Firefly luciferase activity was standardized by Renilla luciferase activity.

### RNA immunoprecipitation (RIP) assay

RIP analysis was performed via EZ-Magna RIP kit (cat no. 17-701; Millipore, Billerica, MA, USA) according to the manufacturer’s instructions. After lysing OV90 and SKOV3 cells in RIP lysis buffer, cell lysates were treated with magnetic beads combined with Ago2 antibody (anti-Ago2) overnight at 4 °C. IgG antibody (anti-IgG) was regarded as a negative control. The precipitated RNAs were purified and measured using qRT-PCR analysis.

### RNA pull-down assay

Biotinylated miR-942-5p (Bio-miR-942-5p) and negative control (Bio-miR-NC) were purchased from GenePharma. OV90 and SKOV3 cells were lysed and then incubated with streptavidin-coated magnetic beads (Invitrogen). Subsequently, the abundance of circ_0015756 was measured using qRT-PCR analysis.

### Xenograft assay

Animal experiments were ratified by the Animal Ethics Committee of Shengjing Hospital of China Medical University. Female BALB/c nude mice (5-week-old) were bought from Beijing Vital River Laboratory Animal Technology Co., Ltd. (Beijing, China) and randomly divided into two groups (n = 5 per group). Lentivirus containing circ_0015756 short hairpin RNA (sh-circ_0015756) or negative control (sh-NC) were purchased from GenePharma and transferred into SKOV3 cells. Then, stably transfected SKOV3 cells (5 × 10^6^) were subcutaneously injected into the flank of mice. Tumor volume was measured every 7 days. After 35 days, the mice were sacrificed and the xenograft tumors were weighed.

### Statistical analysis

All data were expressed as mean ± standard deviation in three independent replicates. The differences were evaluated by Student’s *t*-test or one-way analysis of variance through GraphPad Prism 7 software (GraphPad Inc., La Jolla, CA, USA). The linear relationship among circ_0015756, miR-942-5p and CUL4B was assessed using Spearman’s correlation analysis. When *P*-value < 0.05, the difference was considered statistically significant.

## Results

### Circ_0015756 is up-regulated in OC tissues and cells

**Fig. 1:**
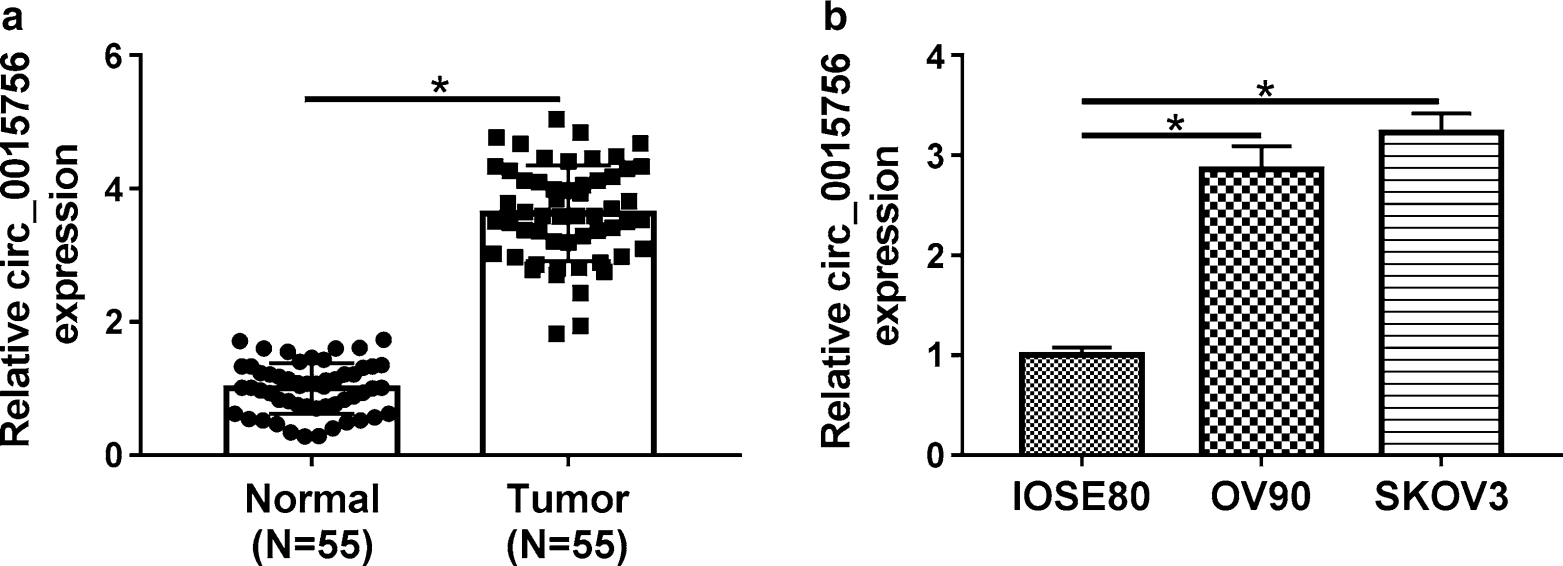
Circ_0015756 is up-regulated in OC tissues and cells. **a** The expression of circ_0015756 in OC tissues (n = 55) and adjacent normal tissues (n = 55) was detected using qRT-PCR. **b** Circ_0015756 level was measured by qRT-PCR in OC cells (OV90 and SKOV3) and normal ovarian epithelial cell line (IOSE80). *P < 0.05

To clarify the role of circ_0015756 in ovarian cancer, we first detected the expression of circ_0015756 in OC tissues. As illustrated in Fig. 1a, circ_0015756 expression in OC tissues was significantly higher than that in normal tissues. Next, we examined the expression of circ_0015756 in OC cell lines and normal ovarian epithelial cell line. As expected, the level of circ_0015756 in OC cell lines (OV90 and SKOV3) was strikingly increased compared with normal ovarian epithelial cell line (IOSE80) (Fig. 1b). These results suggested that circ_0015756 might be an oncogene in ovarian cancer.

### Knockdown of circ_0015756 inhibits the proliferation, migration and invasion of OC cells and induces apoptosis

**Fig. 2:**
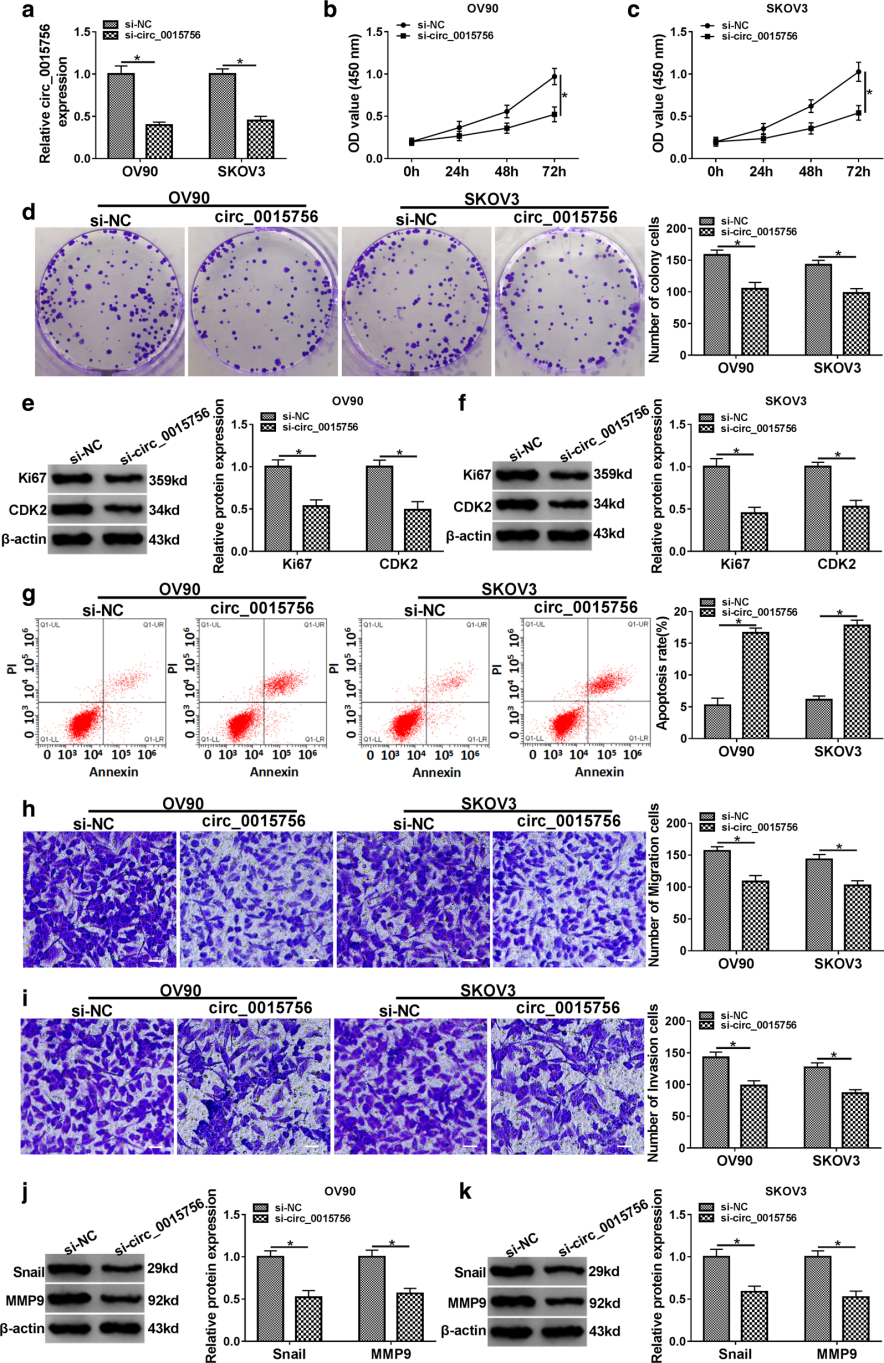
Knockdown of circ_0015756 inhibits the proliferation, migration and invasion of OC cells and induces apoptosis. OV90 and SKOV3 cells were transfected with si-NC or si-circ_0015756, respectively. **a** The knockdown efficiency of circ_0015756 was determined by qRT-PCR. **b**, **c** Cell viability was detected using CCK-8 assay. **d** Cell proliferation ability was estimated by colony formation assay. **e**, **f** The levels of proliferation-related proteins (Ki67 and CDK2) were measured by Western blot. **g** Flow cytometry was used to detect the proportion of apoptotic cells. **h**, **i** Cell migration and invasion capacities were tested by transwell assay (magnification: 100×). **j**, **k** The levels of migration and invasion-associated proteins (Snail and MMP9) were examined by Western blot. *P < 0.05

To further explore the biological function circ_0015756 in OC, OV90 and SKOV3 cells were transfected with si-NC or si-circ_0015756. As shown in Fig. 2a, the knockdown efficiency of circ_0015756 was significant. CCK-8 and colony formation assays showed that circ_0015756 silencing suppressed the proliferation of OV90 and SKOV3 cells compared with the control group (Fig. 2b–d). Consistently, the levels of proliferation-related proteins (Ki67 and CDK2) were markedly reduced in the si-circ_0015756 group compared to the si-NC group (Fig. 2e, f). Flow cytometry suggested that down-regulation of circ_0015756 promoted the apoptosis of OV90 and SKOV3 cells relative to the control group (Fig. 2g). In addition, transwell assay revealed that silence of circ_0015756 inhibited the migration and invasion of OV90 and SKOV3 cells compared with the control group (Fig. 2h, i). Western blot analysis showed that down-regulation of circ_0015756 remarkably decreased the protein levels of Snail and MMP9 compared with the si-NC group (Fig. 2j, k). Overall, these data indicated that silencing of circ_0015756 suppressed OC cell progression.

### Circ_0015756 acts as a miR-942-5p sponge

**Fig. 3:**
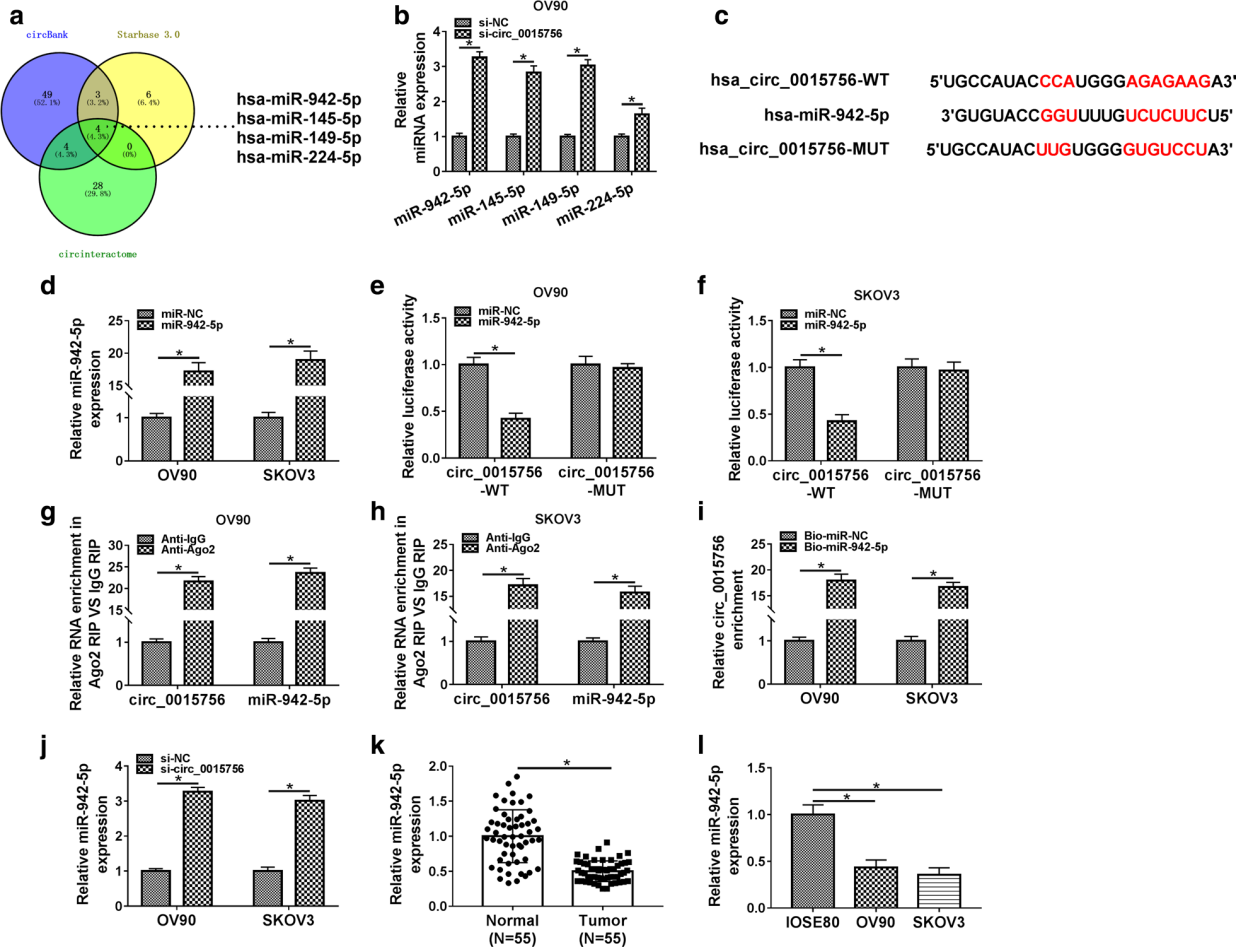
Circ_0015756 acts as a miR-942-5p sponge. **a** Three websites (circBank, Starbase 3.0 and circinteractome) were used to predict potential miRNAs that might bind to circ_0015756. **b** After transfection with si-circ_0015756, the levels of four possible targets (miR-942-5p, miR-145-5p, miR-149-5p and miR-224-5p) were detected. **c** The putative binding site between circ_0015756 and miR-942-5p was depicted by circinteractome. **d** The overexpression efficiency of miR-942-5p was detected by qRT-PCR. The binding relationship between circ_0015756 and miR-942-5p was verified by dual-luciferase reporter assay (**e**, **f**), RIP assay (**g**, **h**) and RNA pull-down assay (**i**) in OV90 and SKOV3 cells. **j** The level of miR-942-5p was determined in OV90 and SKOV3 cells transfected with si-NC or si-circ_0015756. **k**, **l** The expression of miR-942-5p in OC tissues and cells was tested by qRT-PCR. *P < 0.05

Next, we used three prediction websites (circBank, Starbase 3.0 and circinteractome) to predict potential target miRNAs that might bind to circ_0015756 (Fig. 3a). Subsequently, qRT-PCR was used to detect the expression of four possible targets (miR-942-5p, miR-145-5p, miR-149-5p and miR-224-5p) in OV90 cells transfected with si-NC or si-circ_0015756. The results showed that the up-regulation of miR-942-5p was the most significant after circ_0015756 knockdown, so miR-942-5p was selected as a possible target for circ_0015756 (Fig. 3b). The potential binding site between circ_0015756 and miR-942-5p predicted by the circinteractome database was displayed in Fig. 3c. As illustrated in Fig. 3d, the overexpression efficiency of miR-942-5p was significant. To confirm whether circ_0015756 absorbed miR-942-5p, dual-luciferase reporter assay was performed. The results showed that miR-942-5p mimics significantly decreased the luciferase activity of circ_0015756-WT reporter, but did not affect circ_0015756-MUT reporter (Fig. 3e, f). RIP analysis exhibited that circ_0015756 and miR-942-5p were remarkably enriched in the anti-Ago2 group but not the anti-IgG group (Fig. 3g, h). RNA pull-down assay confirmed that circ_0015756 was pulled down by Bio-miR-942-5p instead of Bio-miR-NC (Fig. 3i). In addition, knockdown of circ_0015756 significantly increased the expression of miR-942-5p in OC cells (Fig. 3j). Furthermore, miR-942-5p level was markedly reduced in OC tissues and cells compared with the control group (Fig. 3k, l). Collectively, these data indicated that circ_0015756 negatively regulated miR-942-5p.

### Inhibition of miR-942-5p reverses the effect of circ_0015756 knockdown on OC cell progression

**Fig. 4:**
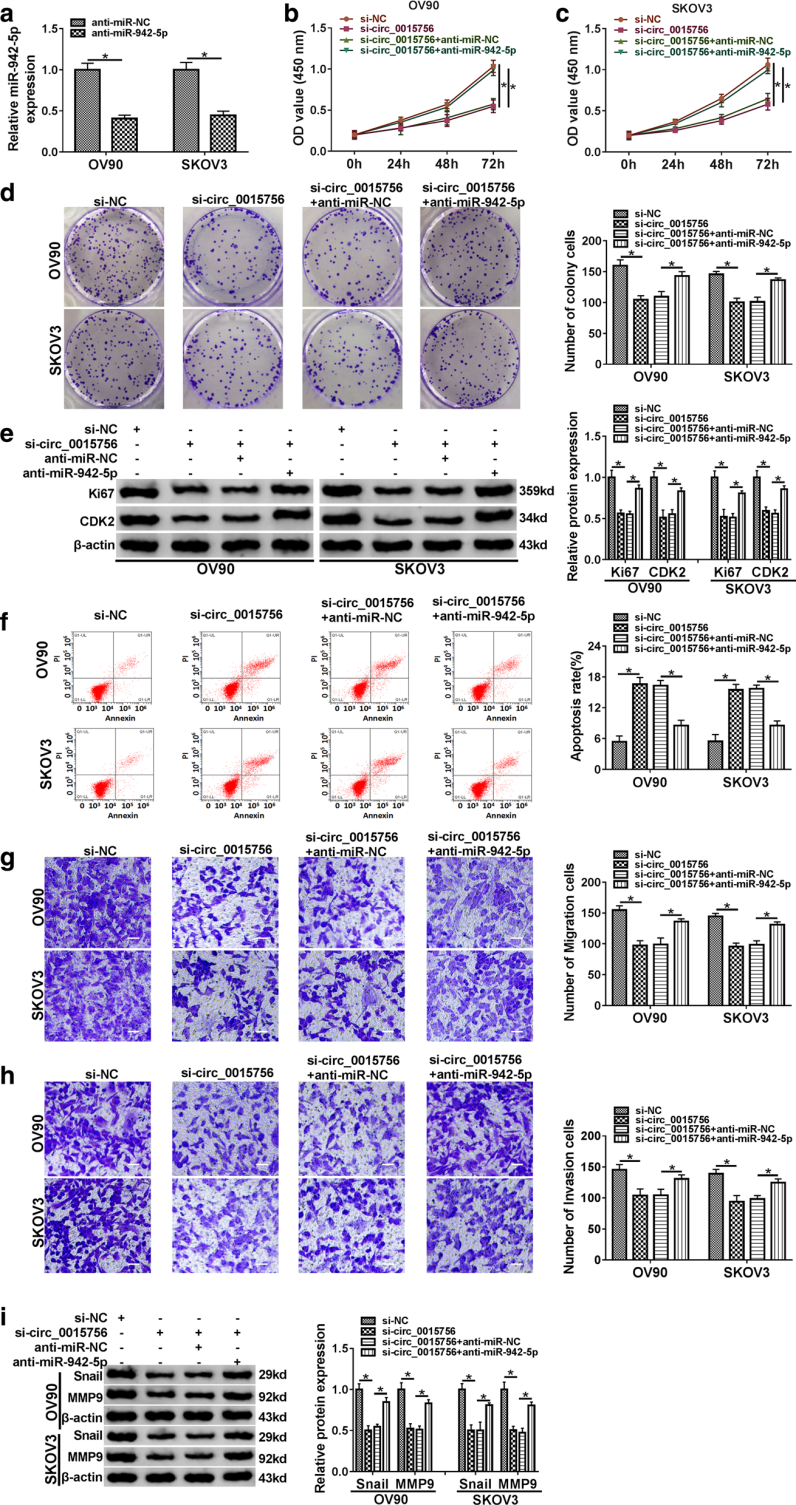
Inhibition of miR-942-5p reverses the effect of circ_0015756 knockdown on OC cell progression. **a** The knockdown efficiency of miR-942-5p was measured by qRT-PCR. **b**–**i** OV90 and SKOV3 cells were introduced with si-NC, si-circ_0015756, si-circ_0015756+ anti-miR-NC or si-circ_0015756+ anti-miR-942-5p, respectively. **b**–**e** Cell proliferation capacity and proliferation-related protein levels were evaluated by CCK-8 assay, colony formation assay and Western blot assay. Cell apoptosis **f**, migration and invasion **g**, **h** and metastasis-related protein levels **i** were detected by flow cytometry, transwell assay and Western blot assay. *P < 0.05

To investigate the role of circ_0015756/miR-942-5p axis in OC cell development, OV90 and SKOV3 cells were transfected with si-circ_0015756 or/and anti-miR-942-5p. Firstly, the inhibition efficiency of anti-miR-942-5p was determined using qRT-PCR (Fig. 4a). CCK-8, colony formation and Western blot assays revealed that circ_0015756 silencing hindered OC cell proliferation, while this effect was partially abolished by down-regulating miR-942-5p (Fig. 4b–e). Flow cytometry and transwell assays indicated that circ_0015756 knockdown induced cell apoptosis and suppressed cell migration and invasion in OV90 and SKOV3 cells, whereas these impacts were reversed after transfection with anti-miR-942-5p (Fig. 4f–h). Moreover, circ_0015756 down-regulation led to a significant decrease in metastasis-related proteins (Snail and MMP9), while this change was partially mitigated by inhibiting miR-942-5p (Fig. 4i). In summary, these data evidenced that circ_0015756 knockdown hindered OC cell progression by regulating miR-942-5p.

### CUL4B is a target of miR-942-5p

**Fig. 5:**
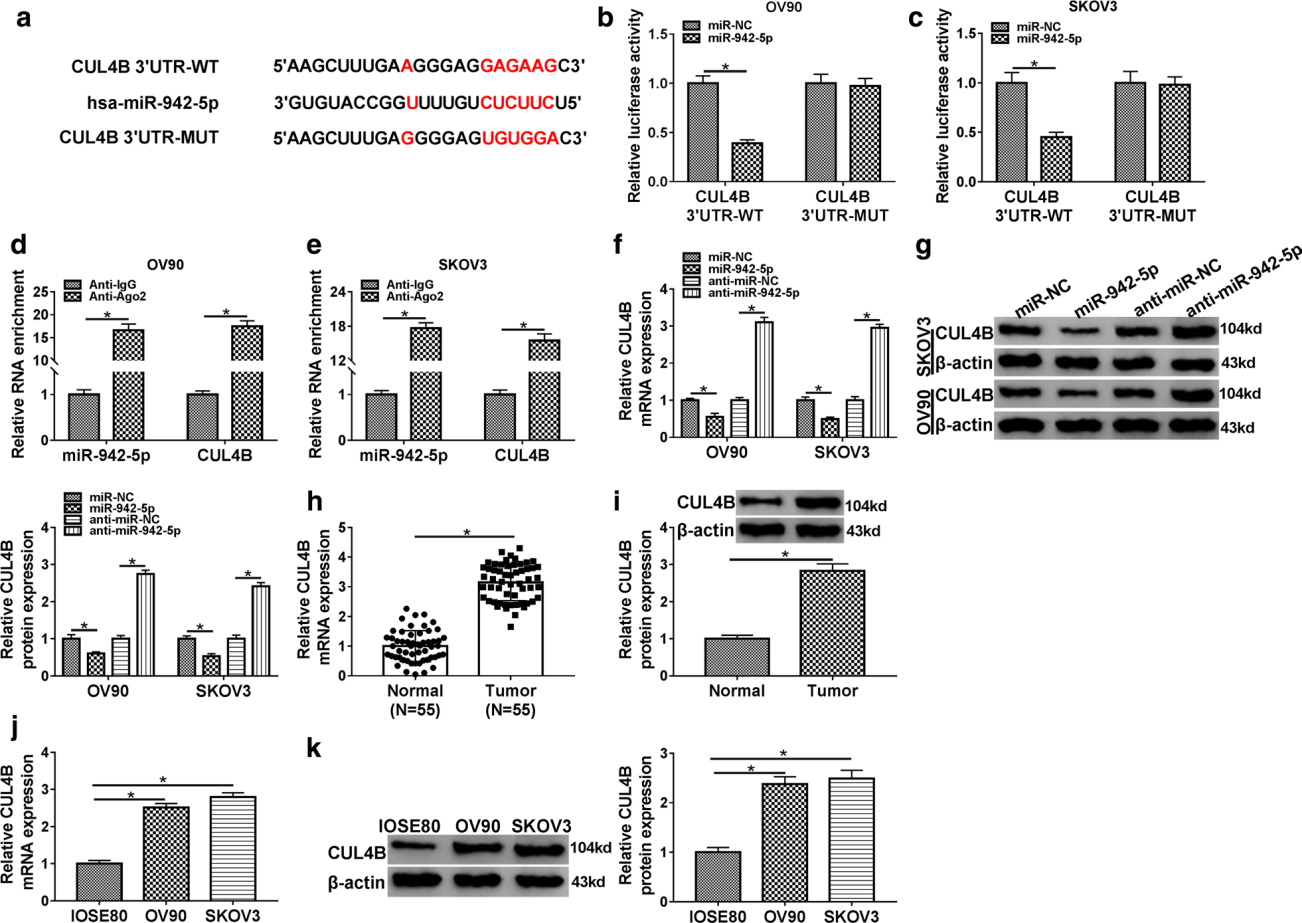
CUL4B is a target of miR-942-5p. **a** The predicted binding site between miR-942-5p and CUL4B 3′UTR was shown. The binding relationship between miR-942-5p and CUL4B 3′UTR was confirmed by dual-luciferase reporter assay (**b**, **c**) and RIP assay (**d**, **e**). **f**, **g** The mRNA and protein levels of CUL4B were measured in OV90 and SKOV3 cells transfected with miR-NC, miR-942-5p, anti-miR-NC or anti-miR-942-5p. **h**, **i** The mRNA and protein levels of CUL4B were examined in OC tissues and normal tissues. **j**, **k** The expression of CUL4B in IOSE80, OV90 and SKOV3 cells was determined by qRT-PCR and Western blot. *P < 0.05

To further explore the downstream molecules of miR-942-5p, Starbase 3.0 database predicted that miR-942-5p and CUL4B 3′UTR had a putative binding site (Fig. 5a). Next, dual-luciferase reporter assay and RIP assay were performed to verify the binding relationship between miR-942-5p and CUL4B. The results exhibited that miR-942-5p mimics remarkably reduced the luciferase activity of CUL4B 3′UTR-WT reporter, but did not affect CUL4B 3′UTR-MUT reporter (Fig. 5b, c). RIP analysis showed that miR-942-5p and CUL4B were significantly enriched in the anti-Ago2 group compared to the anti-IgG group (Fig. 5d, e). Moreover, up-regulation of miR-942-5p markedly inhibited the expression of CUL4B, while down-regulation of miR-942-5p remarkably promoted the expression of CUL4B (Fig. 5f, g). Compared with the normal tissues, CUL4B mRNA and protein levels were strikingly elevated in OC tissues (Fig. 5h, i). Also, the mRNA and protein levels of CUL4B in OV90 and SKOV3 cells were markedly higher than that in IOSE80 cells (Fig. 5j, k). These data indicated that miR-942-5p directly targeted CUL4B.

### CUL4B reverses the effect of miR-942-5p on OC cell progression

**Fig. 6:**
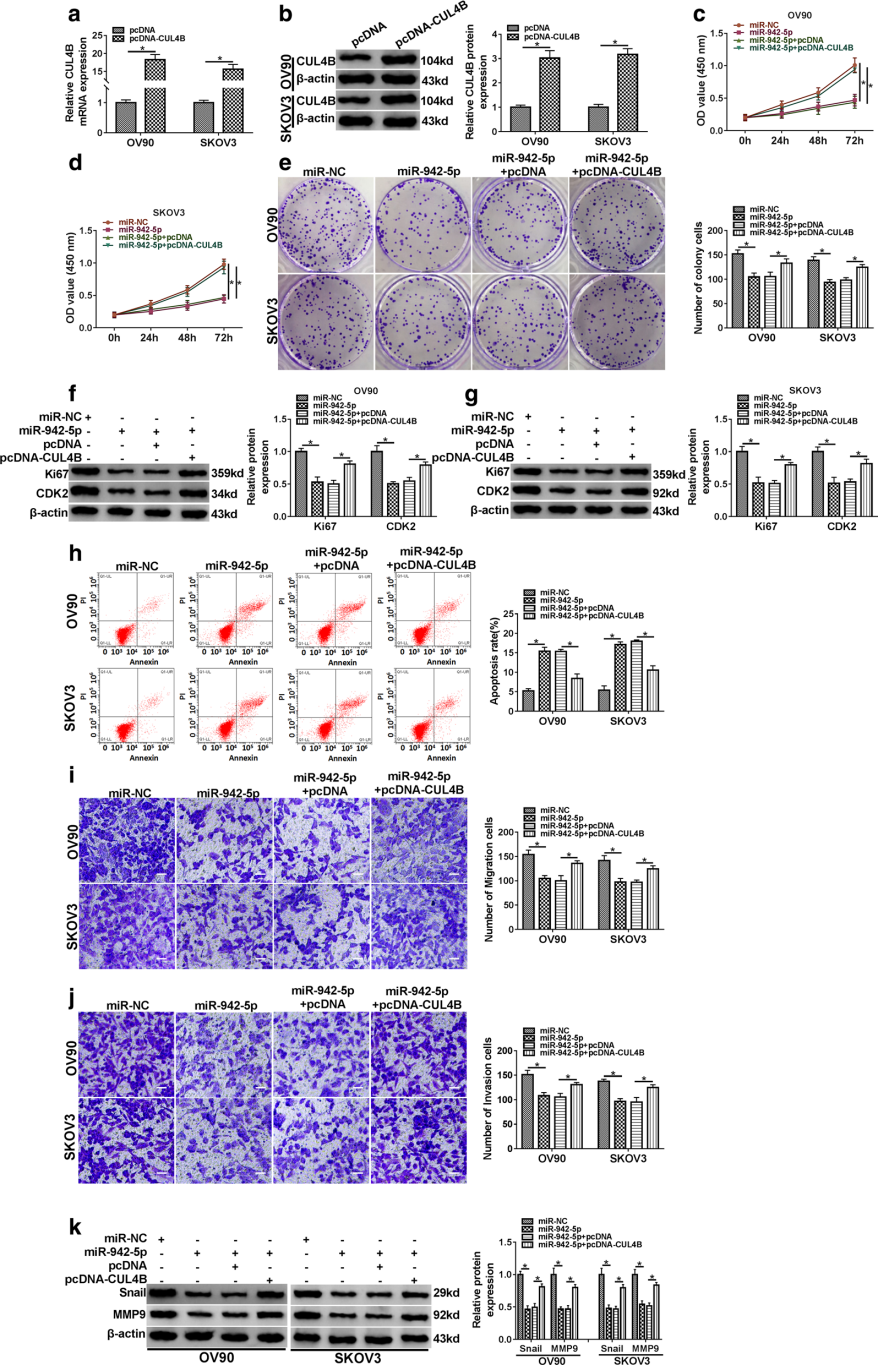
CUL4B reverses the effect of miR-942-5p on OC cell progression. **a**, **b** The overexpression efficiency of CUL4B was detected by qRT-PCR and Western blot. **c**–**k** OV90 and SKOV3 cells were transfected with miR-NC, miR-942-5p, miR-942-5p+ pcDNA or miR-942-5p+ pcDNA-CUL4B. CCK-8, colony formation and Western blot assays were utilized to evaluate cell proliferation capacity (**c**–**e**) and proliferation-related protein levels (**f**, **g**). Flow cytometry, transwell and Western blot assays were used to assess cell apoptosis (**h**), migration and invasion (**i**, **j**) and metastasis-related protein levels (**k**). *P < 0.05

To elucidate whether miR-942-5p targeted CUL4B to affect OC cell development, OV90 and SKOV3 cells were transfected with miR-942-5p mimics or/and pcDNA-CUL4B. As displayed in Fig. 6a, b, the overexpression efficiency of CUL4B was confirmed by qRT-PCR and Western blot. Moreover, introduction of miR-942-5p and pcDNA-CUL4B partially abated the inhibitory effect of miR-942-5p mimics on OC cell proliferation (Fig. 6c–g). Furthermore, up-regulation of miR-942-5p remarkably expedited OC cell apoptosis and suppressed the migration and invasion of OV90 and SKOV3 cells, whereas co-transfection of miR-942-5p and pcDNA-CUL4B reversed these effects (Fig. 6h–k). Overall, these results indicated that miR-942-5p inhibited OC cell progression by modulating CUL4B.

### Circ_0015756 regulates CUL4B expression by targeting miR-942-5p

**Fig. 7:**
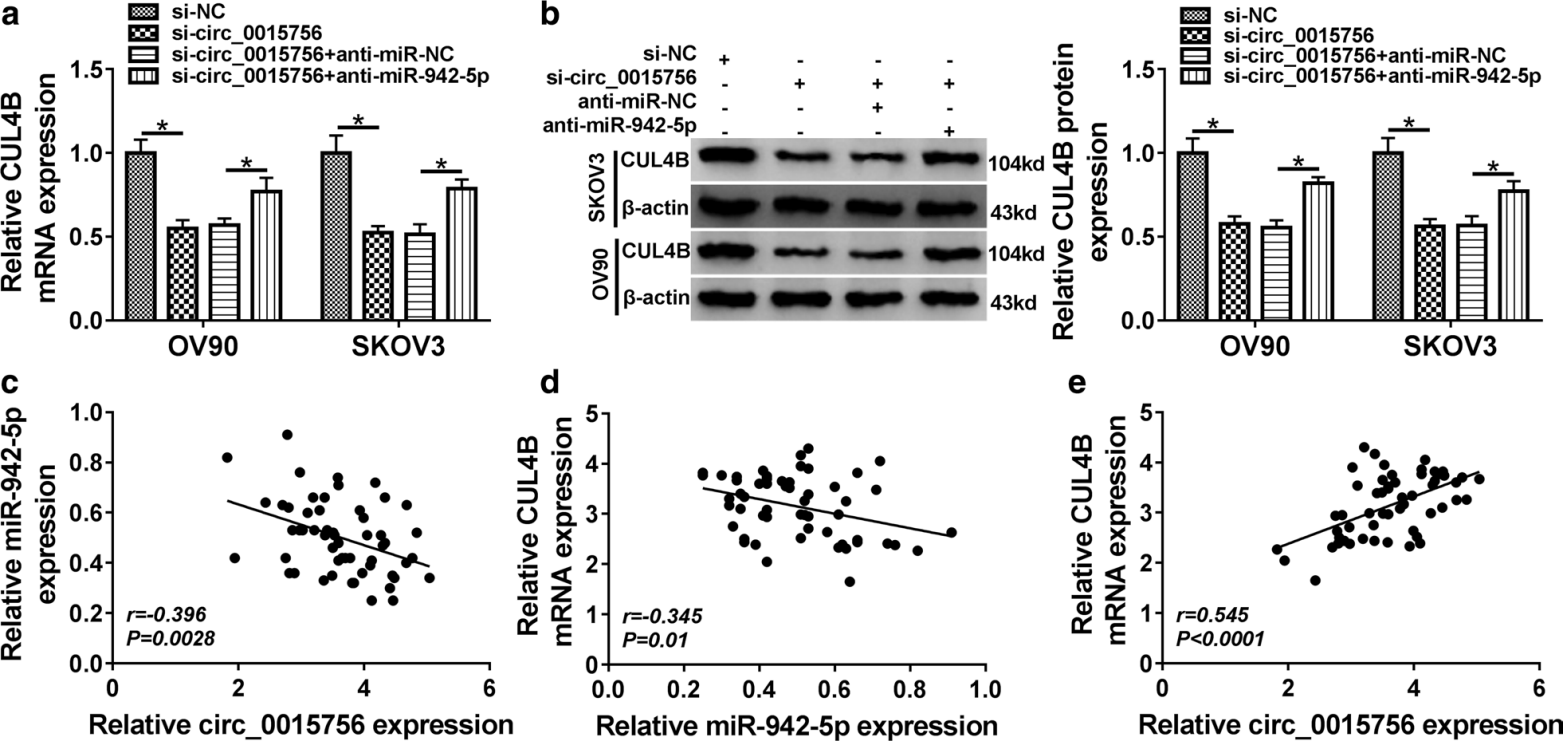
Circ_0015756 regulates CUL4B expression by targeting miR-942-5p. **a**, **b** CUL4B mRNA and protein levels were examined in OV90 and SKOV3 cells transfected with si-NC, si-circ_0015756, si-circ_0015756+ anti-miR-NC or si-circ_0015756+ anti-miR-942-5p. **c**–**e** The correlation among circ_0015756, miR-942-5p and CUL4B was detected by Spearman’s correlation analysis. *P < 0.05

To illuminate the relationship between CUL4B and circ_0015756/miR-942-5p axis, OV90 and SKOV3 cells were transfected with si-NC, si-circ_0015756, si-circ_0015756+ anti-miR-NC or si-circ_0015756+ anti-miR-942-5p. As shown in Fig. 7a, b, co-transfection of si-circ_0015756 and anti-miR-942-5p alleviated the decrease in CUL4B expression caused by circ_0015756 knockdown alone. In addition, Spearman’s correlation analysis showed that miR-942-5p was negatively correlated with circ_0015756 or CUL4B, while circ_0015756 was positively correlated with CUL4B in OC tissues (Fig. 7c–e). These data indicated that circ_0015756 sponged miR-942-5p to up-regulate CUL4B expression.

### Depletion of circ_0015756 hinders OC growth in vivo

**Fig. 8:**
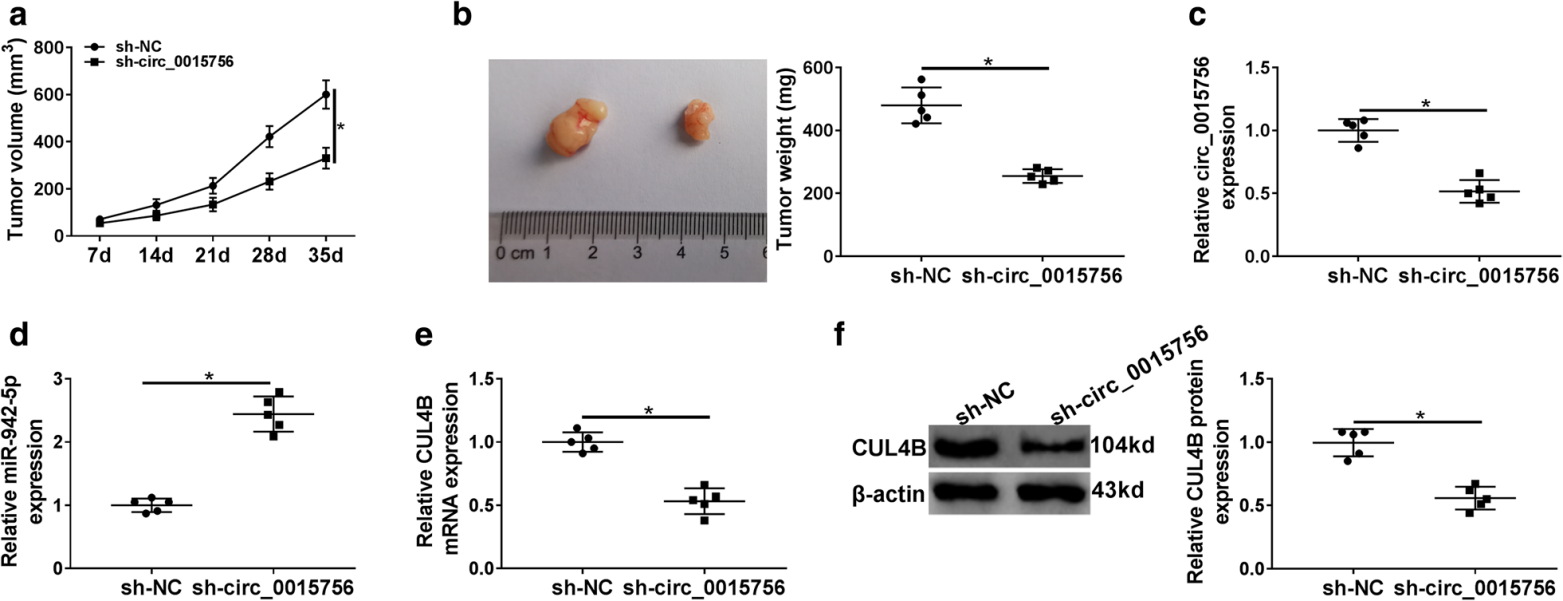
Depletion of circ_0015756 hinders OC growth in vivo. SKOV3 cells containing sh-NC or sh-circ_0015756 were subcutaneously injected into nude mice. **a** Tumor volume was measured every 7 days. **b** The mice were sacrificed 35 days later, and the xenograft tumors were weighed. **c**–**f** The levels of circ_0015756, miR-942-5p and CUL4B were measured by qRT-PCR or Western blot. *P < 0.05

To investigate the role of circ_0015756 in tumorigenesis in vivo, a xenograft model was established. As shown in Fig. 8a, tumor volume was markedly decreased in the sh-circ_0015756 group compared to the sh-NC group. Down-regulation of circ_0015756 remarkably decreased tumor weight compared with the control group (Fig. 8b). Besides, qRT-PCR and Western blot showed that the expression of circ_0015756 and CUL4B was significantly reduced, and the expression of miR-942-5p was strikingly increased in the sh-circ_0015756 group compared to the control group (Fig. 8c–f). These data indicated that circ_0015756 silencing blocked tumor growth in vivo.

## Discussion

Recently, circRNAs have become a hot spot in the field of tumor research. Since most OC patients are diagnosed at the advanced stage, the five-year survival rate is less than 30% [17]. Hence, exploring new treatment strategies and prognostic indicators is crucial for the clinical efficacy of ovarian cancer. Numerous studies have corroborated that circRNAs are abnormally expressed in OC and have diagnostic and prognostic value in OC [18, 19]. In the present research, circ_0015756 level was strikingly elevated in OC tissues and cells. Additionally, silencing of circ_0015756 decelerated cell growth and metastasis in OC cells.

Emerging evidence has highlighted that circRNAs competitively bind miRNAs to act as “molecular sponges”, thereby attenuating the inhibition of miRNAs on mRNA expression [20, 21]. In the current research, the potential targets of circ_0015756 were predicted by bioinformatics analysis. According to the expression of four possible target miRNAs after circ_0015756 knockdown, miR-942-5p was selected as the follow-up research object. Several investigations have shown that circRNAs mediate tumor development through serving as a competing endogenous RNA (ceRNA) for miR-942-5p. For example, circRNA-AKT1 contributed to tumor progression in cervical cancer via decoying miR-942-5p and up-regulating AKT1 [22]. In addition, circ-CEP85L hindered the malignant phenotype of gastric cancer by absorbing miR-942-5p to increase NFKBIA expression [23]. Herein, we unveiled that circ_0015756 sponged miR-942-5p, and circ_0015756 knockdown suppressed OC progression via modulating miR-942-5p.

In terms of mechanism, we also explored the downstream target genes of miR-942-5p. Increasing evidence has manifested that miRNAs affect mRNA expression via binding to mRNA 3′UTR [24]. Zhang et al. [25] disclosed that miR-942-5p overexpression expedited the malignancy of melanoma through inhibition of DKK3. Luo et al. [26] suggested that up-regulation of miR-942-5p attenuated inflammation and apoptosis of septic AKI by repressing FOXO3. In this research, we discovered that miR-942-5p targeted CUL4B and negatively modulated CUL4B.

CUL4B belongs to the scaffold protein of Cullin4B-Ring E3 ligase complex [27]. CUL4B is dysregulated in various tumors and participates in many cellular processes related to tumors [28]. CUL4B functions as a carcinogen in diverse tumors, including bladder cancer [29], colorectal cancer [30] and lung adenocarcinoma [31]. In ovarian cancer, CUL4B was overtly up-regulated in cancer tissues and CUL4B facilitated cancer cell proliferation by mediating CDK2 and CyclinD1 [32]. In addition, many proteins regulate mammalian ovarian development by interacting with numerous binding partners. For example, 14-3-3 (YWHA) protein isoforms are expressed in mouse oocytes and eggs and interact with cell cycle division 25B (CDC25B) [33, 34]. 14-3-3η, the isoform of YWHA protein, regulates the assembly of microtubules by interacting with α-tubulin [35]. In the present research, miR-942-5p targeted CUL4B to suppress OC progression. Furthermore, circ_0015756 elevated CUL4B expression via decoying miR-942-5p.

## Conclusion

In conclusion, circ_0015756 expedited OC progression by sponging miR-942-5p and increasing CUL4B expression. The discovery of circ_0015756/miR-942-5p/CUL4B axis provided a new ceRNA mechanism for OC and a potential therapeutic target for OC treatment. However, the sample size of this study is limited, and a larger sample size is needed to further confirm this conclusion in the future.

## Data Availability

The analyzed data sets generated during the present study are available from the corresponding author on reasonable request.
